# Application of artificial intelligence in the diagnosis and treatment of urinary tumors

**DOI:** 10.3389/fonc.2024.1440626

**Published:** 2024-08-12

**Authors:** Mengying Zhu, Zhichao Gu, Fang Chen, Xi Chen, Yue Wang, Guohua Zhao

**Affiliations:** ^1^ Liaoning University of Traditional Chinese Medicine, Shenyang, China; ^2^ Department of General Surgery, Cancer Hospital of China Medical University, Liaoning Cancer Hospital & Institute, Shenyang, China; ^3^ Department of Gynecology, People's Hospital of Liaoning Province, Shenyang, China

**Keywords:** early diagnosis, treatment, urological tumors, artificial intelligence, medical imaging

## Abstract

Diagnosis and treatment of urological tumors, relying on auxiliary data such as medical imaging, while incorporating individual patient characteristics into treatment selection, has long been a key challenge in clinical medicine. Traditionally, clinicians used extensive experience for decision-making, but recent artificial intelligence (AI) advancements offer new solutions. Machine learning (ML) and deep learning (DL), notably convolutional neural networks (CNNs) in medical image recognition, enable precise tumor diagnosis and treatment. These technologies analyze complex medical image patterns, improving accuracy and efficiency. AI systems, by learning from vast datasets, reveal hidden features, offering reliable diagnostics and personalized treatment plans. Early detection is crucial for tumors like renal cell carcinoma (RCC), bladder cancer (BC), and Prostate Cancer (PCa). AI, coupled with data analysis, improves early detection and reduces misdiagnosis rates, enhancing treatment precision. AI’s application in urological tumors is a research focus, promising a vital role in urological surgery with improved patient outcomes. This paper examines ML, DL in urological tumors, and AI’s role in clinical decisions, providing insights for future AI applications in urological surgery.

## 1 Introduction

AI refers to the capability of simulating human intelligence through computer technology (1, 2). It involves empowering computer systems to execute tasks akin to human intelligence, including language comprehension, learning, logical reasoning, problem-solving, and perception. It has reshaped and innovated medical systems by drawing on principles from fields such as mathematics, logic, computing, and biology. This innovation has not only enhanced the ability of physicians to perform medical tasks but also helped medical technologists overcome previously laborious challenges. With the progress of ML and DL, AI systems have the capacity to analyze extensive medical data, encompassing patient records and imaging scans, offering valuable insights for diagnosis, treatment planning, and patient care (3). Moreover, AI-powered medical tools and systems, such as decision support and diagnostic algorithms systems, assist healthcare professionals in making accurate and timely clinical decisions (4). This collaboration not only streamlines processes but also improves patient outcomes, holding tremendous potential to change the landscape of medical practice, research, and education, ushering in an era of precision medicine and data-driven medical innovation.

ML is a vital facet of AI, involving the creation and implementation of adaptive algorithms that are pivotal in data analysis and pattern recognition (5). Through research and development, endeavors have been made to enable computers to learn from experience and construct mathematical models capable of solving problems. These models can encapsulate acquired domain knowledge and make accurate predictions on new data. The application domains of ML are extensive, covering areas such as data analysis, imaging technologies, healthcare, and diagnostics (6). Through ML, personalized healthcare can be achieved, enabling accurate prediction and diagnosis of diseases such as RCC, BC, and PCa (7). The development of these technologies has brought significant advancements to the field of healthcare, providing more effective solutions for human health. In summary, the development of ML presents vast prospects for the AI field, showcasing immense potential across various domains.

The rapid development of DL continues to drive the advancement of AI. One of the key characteristics of DL is its emphasis on feature learning, enabling computers to automatically learn useful features and representations from raw data, thereby enabling them to discover complex patterns within vast datasets (8). Unlike traditional ML approaches, DL integrates feature extraction and task completion into a unified framework, allowing both aspects to improve simultaneously during continuous training (9). In the field of medical imaging, DL is primarily implemented through CNNs, a powerful algorithm particularly suited for image learning and other structured data tasks (10, 11). Leveraging DL techniques, medical images can be rapidly and accurately analyzed and interpreted, providing clinicians with powerful tools to aid in more precise disease diagnosis, treatment planning, and treatment monitoring (3). The development of this technology not only enhances the efficiency of healthcare delivery but also improves the diagnostic experience and treatment outcomes for patients.

The extensive use of ML and DL techniques within artificial intelligence, notably the advancements made by CNNs in recognizing medical images, has facilitated more accurate and personalized diagnosis and treatment of genitourinary tumors. These technologies can handle the complex structures and patterns present in medical images, thereby improving diagnostic accuracy and efficiency. By learning and analyzing large datasets, AI systems can uncover hidden features and patterns within images (12), providing clinicians with more reliable diagnostic recommendations and personalized treatment plans. Early diagnosis and treatment are crucial, especially for genitourinary tumors such as RCC, BC, and PCa. Through the utilization of ML and DL techniques combined with the analysis of large-scale data, the early detection rate of these tumors can be improved, reducing misdiagnosis rates and offering patients more precise treatment options (13). As a result, the utilization of artificial intelligence for diagnosing and treating genitourinary tumors has become a focal area of research in medicine. With ongoing research and technological advancements, it is anticipated that artificial intelligence will increasingly influence urology, enhancing medical services and treatment outcomes for patients.

## 2 The application of AI in RCC

RCC ranks seventh among cancers affecting men and ninth among those affecting women worldwide. The annual global incidence is estimated at about 209,000 new cases, leading to approximately 102,000 deaths annually. Furthermore, global incidence and mortality rates are rising by approximately 2-3% per decade (14). Addressing this challenge, recent years have seen the emergence of AI techniques in RCC research. AI can analyze extensive imaging and clinical datasets to reveal patterns and correlations, offering comprehensive support and insights for RCC diagnosis, treatment planning, and adjunctive therapy (15). The application of this emerging technology offers hope for improving the survival rates and treatment outcomes of RCC patients, while also providing new directions and opportunities for future research and clinical practice.

### 2.1 The application of AI in the diagnosis of RCC

Advanced AI algorithms can precisely categorize renal masses as malignant or benign, significantly aiding in disease diagnosis and treatment decisions. Xi et al. investigated 1,162 cases of renal lesions from multiple centers, identifying 655 cases as malignant lesions and 507 cases as benign lesions. Compared with the baseline zero rule algorithm, expert averages, and radiological models, the integrated DL model demonstrated superior test accuracy, sensitivity, and specificity (16). Kunapuli et al. (15) proposed RFGB as a promising clinical decision support tool for the diagnosis of renal masses. Unlike other studies, the patient population in this research better mirrors that of real-world clinical practice, enhancing the relevance of clinical decision support through the use of multiphase contrast-enhanced CT images and diverse ML algorithms to develop effective and interpretable models. Oberai and colleagues devised a CNN classifier using multiphase CECT images to diagnose solid-enhancing malignant renal masses, achieving an accuracy of 78%, a sensitivity of 70%, and an area under the curve (AUC) of 0.82 (17).

Accurate differentiation among RCC subtypes is vital, particularly the three main types (clear cell, papillary, and chromophobe), which collectively constitute more than 90% of RCC cases. Uhm et al. (18) proposed an end-to-end DL model to differentiate between five major histological subtypes of renal tumors, including both benign and malignant types (oncocytoma, acute myeloid leukemia, clear cell RCCs, papillary RCCs, and chromophobe RCCs), using multiphase CT imaging. The study utilized CT data from 308 patients who had undergone nephrectomy for renal tumors to train and validate the model. The model demonstrated superior performance compared to radiologists across most subtypes, achieving an AUC of 0.889. Kocak et al (19). utilized machine learning to analyze imaging data of RCC with the aim of developing and validating a quantitative computed tomography textural analysis (qCT-TA) model. The study included internal validation data from 68 RCC patients and external validation data from 26 cases sourced from the TCGA public database. They extracted 275 textural features from non-enhanced and corticomedullary phase CT images. Reproducibility analysis involved three radiologists, and feature selection utilized classifier-based wrapper methods. The model underwent optimization through nested cross-validation, combining artificial neural networks (ANNs), support vector machines (SVMs), and other algorithms to improve generalizability. The research addressed two classification tasks: papillary RCC from chromophobe RCC, and distinguishing non-clear cell RCC from clear cell RCC. The main performance metric evaluated was the Matthews correlation coefficient (MCC). The ML-based qCT-TA showed effective differentiation between non-clear cell RCC and clear cell RCC, achieving external validation accuracies of 84.6% for accuracy, 69.2% for sensitivity, and 100% for specificity. In another investigation, Kocak et al. (20) studied 45 patients diagnosed with clear cell RCCs, categorized into 29 cases without PBRM1 mutation and 16 cases with PBRM1 mutation. Texture features were extracted from contrast-enhanced CT images during the corticomedullary phase using an open-source software package. Among the 828 texture features initially extracted, 759 demonstrated excellent reproducibility. Using 10 selected features, the ANNs algorithm accurately classified 88.2% of clear cell RCC cases based on PBRM1 mutation status. Similarly, the RF algorithm achieved a classification accuracy of 95.0% with five selected features. These results suggest that ML-based high-dimensional qCT-TA shows promise as a viable method to predict PBRM1 mutation status in clear cell RCC patients (20). Wen et al. explored both the staging and grading of clear cell RCCs in their study. They analyzed clinical and pathological data from 878 patients diagnosed with renal clear cell carcinoma, employing DL algorithms to predict both pathological staging and grading based on preoperative clinical variables. For tumor staging, the BiLSTM, CNN-BiLSTM, and CNN-BiGRU models achieved respective AUC values of 0.933, 0.947, and 0.948. In terms of tumor grading, the corresponding AUC values were 0.754, 0.720, and 0.770 (21). Ding et al. extracted texture features from CT images of the largest renal mass area during both the corticomedullary and nephrographic phases. They computed a texture score for each patient and developed a model based on these scores (22). In an independent validation cohort of 92 ccRCC cases, the predictive model’s performance was evaluated and compared. The texture score-based model exhibited superior discriminative capability for distinguishing between high-grade and low-grade ccRCC, irrespective of the inclusion of non-texture features (P < 0.05). This study suggests that a texture score-based model could facilitate preoperative differentiation between high-grade and low-grade ccRCC.

### 2.2 The application of AI in the treatment of RCC

Alexander Kutikov introduced the R.E.N.A.L. nephrometry score to standardize reporting on renal tumor characteristics, essential for treatment decisions and meaningful comparisons. This scoring system assesses five key anatomical features of renal masses using computed tomography or magnetic resonance imaging. Four components are scored on a scale of 1 to 3, while the fifth identifies the mass’s anterior or posterior position relative to the kidney’s coronal plane. This systematic approach offers clinicians a valuable tool for comprehensive characterization of renal tumors in clinical practice (23). In postoperative care, the recording of intraoperative parameters and events is crucial. This data can aid medical teams in predicting the risk of postoperative complications. For instance, unstable events during surgery, changes in patient vital signs, and the duration of surgery may all serve as important indicators of postoperative complications. Mathieu et al (24). retrospectively analyzed data from 240 patients undergoing robot-assisted laparoscopic partial nephrectomy (RALPN). After analyzing patient age, body mass index, anticoagulant therapy, tumor size, and surgical duration, researchers identified a correlation between RALPN and a 30% likelihood of postoperative complications. Regarding RALPN, artificial intelligence plays a crucial role in evaluating how factors like surgeon experience, blood loss, and extent of system resection influence the occurrence of complications. Zhao et al. (25) conducted a study aimed at developing an accurate prediction model for the duration of robot-assisted surgery (RAS) using ML techniques. This model takes specific preoperative patient parameters and surgery-related factors (including tumor location and patient comorbidities) as input data. Compared to the baseline model, all ML models reduced the mean root mean square error (RMSE). Notably, the enhanced regression tree exhibited the lowest average RMSE, significantly outperforming the baseline model, with the accuracy of planned cases increasing from 35% to over 50%. This study emphasizes the potential of various ML methods to improve the accuracy of predicting the duration of RAS cases.

Developing a model to predict the survival rate, recurrence risk, or other outcomes of kidney cancer patients can assist clinicians in devising more personalized treatment plans and offering more precise patient consultations. Utilizing multi-gene expression and biomarkers allows for the evaluation of survival rates and prognoses in kidney cancer patients. Researchers conducted a retrospective study examining radiomic features extracted from CT images to investigate their correlation with the tumor microenvironment. The study encompassed 78 patients, analyzing radiomic features from CT scans, specifically exploring CD8 T cell infiltration and programmed death-ligand 1 (PDL1) expression. Findings indicated that CT radiomic features successfully differentiated between tumors with high infiltration and those without, as well as between tumors positive and negative for PDL1 expression (26). This may hold potential for predicting prognosis and treatment response. Li et al. (27) developed a risk scoring model based on 15 genes using gene expression analysis, machine learning (random forest variable selection), and Cox regression. They validated the model using data from TCGA and E-MTAB-1980 datasets. Their findings revealed that patients classified into the high-risk group exhibited markedly lower overall survival (OS) rates compared to those in the low-risk group. Interestingly, the risk score was independent of age and gender but showed significant correlations with hemoglobin levels, primary tumor size, and grade. Importantly, the prognostic value of the risk score remained consistent regardless of whether patients underwent radiotherapy. Buchner et al. (28) assembled data from 175 RCC patients commencing systemic therapy at a single-center database. Factors including age, gender, body mass index, type of systemic therapy, and number of metastatic lesions were inputted into an ANNs, with a logistic regression model constructed using the same variables. Results demonstrated that the ANNs achieved an overall accuracy of 95%, markedly surpassing the logistic regression model. The study underscores the efficacy of predictive models developed using artificial intelligence techniques, highlighting their potential to offer more diverse and personalized treatment strategies for patients with renal cancer.

## 3 The application of AI in BC

BC ranks sixth among cancers diagnosed in the United States, with males ranking it fourth (29). Worldwide, it is the ninth most prevalent malignancy, with around 430,000 new cases reported each year (30). Non-muscle-invasive bladder cancer (NMIBC) represents about 75% of initial diagnoses (31) and demonstrates frequent recurrence and progression in intermediate to high-risk cases. Due to its propensity for recurrence, patients often necessitate regular surveillance and intervention. The objective of this therapeutic approach is to promptly detect and manage any potential recurrence or progression, thereby improving patient survival rates and quality of life.

### 3.1 The application of AI in the diagnosis of BC

Originally, diagnostic cystoscopy was one of the most commonly used methods for detecting BC, especially when evaluating hematuria (32). In recent years, DL has demonstrated promising potential in the diagnosis of BC. Researchers have achieved a series of positive results by utilizing DL algorithms to analyze various medical data, including cystoscopy examinations, urine cytology, and imaging. Shkolyar et al. (33) created CystoNet, an image analysis platform utilizing CNNs, and evaluated videos from 54 patients undergoing transurethral resection of bladder tumor (TURBT) or clinical flexible cystoscopy. The CystoNet algorithm successfully detected bladder cancer in the validation dataset, achieving per-frame sensitivity and specificity of 90.9% and 98.6%, respectively, and per-tumor sensitivity of 95.5%.In their research, Ikeda et al. (34) developed and rigorously tested a pre-trained CNNs model trained on ImageNet. They evaluated a total of 335 normal images and 87 tumor images, achieving 78 true positives and 315 true negatives. The model also generated 20 false positives and 9 false negatives. According to ROC curve analysis, the model exhibited an AUC of 0.98, with a maximum Youden index of 0.837. The sensitivity of the model was determined to be 89.7%, and its specificity was 94.0%. Zhang et al. (35) conducted a study where radiomic features were extracted from regions of interest (ROIs) containing bladder cancer (BC) tissue in diffusion-weighted imaging (DWI) and corresponding apparent diffusion coefficient (ADC) maps obtained from 3.0 Tesla magnetic resonance imaging (MRI). Their approach involved histogram and gray-level co-occurrence matrix (GLCM) analyses, resulting in 102 features extracted per ROI. Initially identifying 47 candidate features, they narrowed it down to a subset of 22 optimal features. Using a SVM classifier with this subset, they achieved superior performance in BC grading, with an area under the curve (AUC) of 0.861, accuracy of 82.9%, sensitivity of 78.4%, and specificity of 87.1%. In one study, Lorencin (36) and his colleagues employed a combination of multilayer perceptron (MLP) and DL CNNs for the detection of urinary BC. A dataset comprising 1997 images of BC and 986 images of non-cancerous tissue was utilized for training and testing purposes. Results demonstrated that utilizing images preprocessed with Laplacian edge detector achieved an AUC value as high as 0.99 when employing MLP for training and testing. Furthermore, comparison of different image sizes revealed optimal performance with 50×50 and 100×100 images.

### 3.2 The application of AI in the treatment of BC

In urology, CT scans, MRI, and ultrasound are essential imaging modalities, crucial for diagnosing and treating BC. Each technique serves distinct roles: CT scans offer detailed three-dimensional images with high resolution, MRI excels in visualizing soft tissues, and ultrasound provides a convenient, non-invasive method for examinations. These tools play critical roles from initial diagnosis and staging to monitoring treatment responses and disease progression in BC (37). However, despite significant advances in these technologies, there are still limitations such as operator dependence and subjectivity in data interpretation. In this context, the introduction of AI technologies has brought new possibilities to imaging techniques. By training algorithms to analyze extensive imaging datasets, AI can swiftly and accurately detect potential lesions, aiding physicians in making precise diagnoses and treatment plans (38, 39). Cha et al. developed a computerized decision support system (CDSS-T) using CT imaging to evaluate treatment response in muscle-invasive BC. They applied CDSS-T to analyze 123 patients with 157 lesions of muscle-invasive BC. This system integrates deep learning CNNs and radiomic features to distinguish between lesions showing complete response to neoadjuvant therapy and those that do not. Analysis of pre- and post-chemotherapy CT scans indicated that CDSS-T achieved an average AUC of 0.80 for assessing pathological T0 disease, compared to 0.74 for physicians without CDSS-T and 0.77 for those using CDSS-T. These results suggest that CDSS-T enhances physicians’ ability to identify complete responses to neoadjuvant chemotherapy in muscle-invasive BC (40). Segmenting the bladder wall from MRI images is crucial for early detection and supportive diagnosis of bladder tumors. Li et al. (41) introduced an automated segmentation technique utilizing DL and anatomical constraints. Initially, they employed an autoencoder to extract concise feature representations of the bladder wall from MRI and labeled images, effectively capturing anatomical and semantic details. Subsequently, these constraints were integrated into an enhanced residual network to enhance segmentation accuracy. Experimental findings from 1092 MRI images illustrated that their method outperformed existing approaches, achieving a Dice similarity coefficient (DSC) of 85.48% (41).

Artificial intelligence has also played a crucial role in personalized treatment. By analyzing extensive clinical data and patient case histories, ML models can predict treatment responses and survival rates, thereby supporting clinicians in devising more precise treatment plans. Buchner et al. (42) conducted a study to assess the use of ANNs in stratifying the risk of BC patients undergoing radical cystectomy (RC) based on readily available procedural parameters. They utilized data from 2111 patients to train ANNs to predict tumor recurrence, cancer-specific mortality (CSM), and all-cause mortality (ACD). The study revealed that the optimal ANNs achieved accuracies of 74%, 76%, and 69% for predicting tumor recurrence, CSM, and ACD, respectively. Notably, lymph node status emerged as a crucial factor in the decision-making process of the networks. Compared to Cox proportional hazards regression models, ANNs demonstrated superior predictive accuracies for recurrence, CSM, and ACD, showing improvements of 1.6% (p = 0.247), 4.7% (p < 0.001), and 3.5% (p = 0.007), respectively. In 2021, Bhambhvani et al. (43) conducted a study using a dataset comprising 161,227 patients, employing an artificial ANNs model trained and validated on an 80/20 split of the dataset. They also developed a multivariable Cox Proportional Hazards (CPH) model that incorporated variables such as age, gender, race, grade, SEER stage, tumor size, lymph node involvement, extent of spread, and surgical intervention for predictive purposes. The study focused on evaluating 5-year Disease-Specific Survival (DSS) and 5-year OS as primary endpoints. Results indicated that the ANNs models achieved AUCs of 0.81 for OS and 0.80 for DSS, whereas the CPH models yielded AUCs of 0.70 for OS and 0.81 for DSS (43). Notably, the ANNs model demonstrated superior accuracy in predicting OS, highlighting the potential of machine learning algorithms to advance prognostic capabilities in bladder cancer. Despite the demonstrated reliability of AI models based on image analysis for predicting the prognosis of BCa, several challenges still need to be addressed. One major challenge is the issue of parameter standardization. Different studies may use varying parameters, leading to inconsistency in results and making it difficult to compare and apply the models. Another hurdle involves the collection and annotation of data. Effective training and validation of AI models necessitate substantial quantities of high-quality data. Nevertheless, existing datasets are often constrained, particularly concerning comprehensive long-term follow-up and detailed clinical information. Additionally, data annotation requires the involvement of skilled medical professionals, demanding significant time and resources. Despite these challenges, once resolved, AI models will be able to provide powerful tools for predicting the prognosis of BCa patients preoperatively, intraoperatively, and postoperatively.

## 4 The application of AI in PCa

PCa is a common and deadly malignant tumor worldwide. There are approximately 1.2 million new cases annually (44), with high incidence and relatively high mortality rates. Diagnosis of this cancer typically relies on transrectal prostate biopsy, which, although providing accurate pathological diagnosis, carries potential complications such as bleeding, infection, and urinary retention. These risks may contribute to overdiagnosis and overtreatment, particularly in low-risk patients. To mitigate these concerns, clinicians must conduct comprehensive prognostic assessments utilizing the latest diagnostic techniques and tools to accurately determine tumor staging and risk stratification. Such assessments facilitate the development of personalized treatment plans, ensuring patients receive optimal therapy while minimizing unnecessary interventions and potential complications. Current research endeavors are focused on identifying more effective and precise diagnostic modalities to enhance treatment efficacy and patient survival outcomes in PCa management.

### 4.1 The application of AI in the diagnosis of PCa

Ishioka et al. (45) reviewed data from 335 patients with prostate-specific antigen levels <20 ng/mL who underwent MRI and extended systematic prostate biopsy. They developed a computer-aided diagnosis (CAD) algorithm using deep CNNs, trained on MR images labeled “cancer” or “non-cancer” based on biopsy results. The CAD algorithm, achieving high diagnostic accuracy in two evaluation datasets, was then applied to a previously unassessed independent dataset. ROC curve analysis demonstrated the CNNs-based CAD system’s capability to automate prostate cancer detection through MRI with over 90% accuracy, sensitivity, and specificity. This technology shows potential for enhancing standardization and consistency in clinical practice (45).

The Gleason scoring system (46), developed in the 1960s to 1970s, is essential for predicting tumor behavior and categorizing tumors into low, intermediate, and high-risk groups. However, despite its critical role in prognosis and patient management, the scoring conducted by pathologists is subjective and prone to variability both between different observers and within the same observer. Studies have shown a discordance in Gleason scores ranging from 30% to 53% (47). Bulten et al (48). developed a DL system for grading prostate biopsies based on the Gleason scoring system. Utilizing 5759 biopsy samples from 1243 patients, they found a high concordance between the system and the reference standard, with a strong performance at the clinical decision threshold. Results from observer experiments indicated that the DL system outperformed the group (median kappa 0.819) with a score of kappa 0.854, and demonstrated superior performance among 15 pathologist observers. This study provides evidence that the automated DL system may have a positive impact on PCa diagnosis in the context of Gleason grading (49). Karimi et al. devised a DL classification method and employed data augmentation strategies to enhance the grading accuracy of PCa in histopathology images, even with limited data. Their technique involves integrating predictions from three distinct CNNs, each trained on patches of different sizes. Their approach yielded promising outcomes, achieving a 92% accuracy in distinguishing cancerous from benign patches and 90% accuracy in distinguishing low-grade (Gleason grade 3) from high-grade (Gleason grades 4 and 5) patches (48).

### 4.2 The application of AI in the treatment of PCa

Patients diagnosed with PCa often experience confusion regarding the treatment options available to them. Establishing a system to assist patients in understanding various therapies can alleviate anxiety and enhance satisfaction. Auffenberg et al. (50) created a predictive model employing the random forest machine learning technique, validated with 7543 men diagnosed with PCa. The personalized predictions for patients in the validation group exhibited high accuracy (AUC 0.81). This model aims to help newly diagnosed men with prostate cancer explore anticipated treatment strategies for comparable cases, potentially reducing pre-treatment concerns. Abdollah et al (51). collected MRI scans from 33 patients diagnosed with Pca according to established standards. Changes in ADC values were employed to evaluate the response to intensity-modulated radiation therapy (IMRT), dividing patients into responsive and non-responsive groups. The study identified 15 patients (45% of the total) categorized as responsive. Importantly, significant differences in two T2-weighted and fifteen ADC radiomic features were observed between the responsive and non-responsive groups, underscoring their predictive value for treatment response. Ultimately, the study discovered a multitude of features within pre-treatment MRI images that hold potential for predicting early treatment outcomes, offering valuable insights for personalized treatment strategies. In 2021, researchers at Google Health led by Wulczyn et al. (52) devised a deep learning model to assess the risk of prostate cancer-specific mortality. They tested the model’s efficacy in stratifying patient risk using a retrospective cohort comprising 2807 cases of prostatectomy from a European center. The AI’s C-index was 0.82, demonstrating the efficacy of the artificial intelligence system in accurately assessing the risk of PCa-specific mortality. Their research showcased the potential of AI in enhancing clinical decision-making and patient care in this critical area of oncology. Hung et al. (53) developed a DL model to forecast urinary incontinence recovery following radical prostatectomy (RARP), aimed at assessing the postoperative outlook for PCa patients. Through statistical analyses including Kruskal-Wallis, chi-square, and Fisher exact tests on historical cases, they determined that approximately 79% of the 79 patients experienced urinary incontinence at a median of 126 days after surgery. The DL This study demonstrated that combining clinical pathology data with APM was more effective in predicting postoperative urinary incontinence than using clinical pathology data or APM alone. Hakyung Lim (54) and colleagues developed an AI-powered Decision Support System (DSS) to optimize treatment sequencing for castration-resistant prostate cancer (CRPC). They analyzed clinical and pathological data from 801 CRPC patients, using the Cox proportional hazards regression model with Extreme Gradient Boosting (XGB). Their study assessed how abiraterone acetate, cabazitaxel, docetaxel, and enzalutamide influence cancer-specific mortality (CSM) and overall mortality (OM). The findings showed that the XGB model outperformed RSF and Cox models in predicting CSM and OM. This online DSS tool aims to assist clinicians and patients in making well-informed decisions regarding CRPC treatment sequencing. Bumjin Lim (55) and colleagues conducted an external validation study to evaluate the SCaP survival calculator’s performance. This tool uses a long short-term memory artificial neural network to predict survival outcomes in prostate cancer patients based on their initial treatment. Analyzing clinical data from 4,415 patients, the study assessed its accuracy in predicting CRPC-free survival, cancer-specific survival (CSS), and OS. Results showed the validation cohort exhibited superior AUC values compared to the development cohort, affirming the SCaP survival calculator’s robust performance in predicting survival outcomes. This study underscores its role as a reliable tool for guiding treatment decisions and predicting survival outcomes in newly diagnosed PCa patients. Kyo Chul Koo (56) and his team studied 7,267 PCa patients, employing diverse ANNs models (MLP, MLP-N, LSTM) to forecast CRPC progression-free survival, CSS, and OS across varied initial treatments. Their findings indicated superior performance of ANN models over traditional Cox models, particularly the LSTM model, which delivered personalized survival predictions. This research highlights the potential of LSTM ANN models to tailor survival forecasts based on initial treatment strategies for PCa. The above study suggests that AI has promising prospects in predicting surgery, radiotherapy, prognosis, and other aspects of PCa.

## 5 Summary and outlook

In recent times, notable progress has been achieved in applying artificial intelligence within the realm of urologic oncology. Through techniques such as DL and ML, researchers have leveraged big data analytics to improve the accuracy of urologic tumor diagnosis, predict disease progression and treatment response, and provide more precise treatment recommendations for clinical practitioners. However, AI in urologic oncology also faces several challenges. Initially, the quality and annotation of data are pivotal for training models; however, obtaining high-quality medical data and accurate annotations remains intricate and time-intensive. Secondly, the interpretability of AI models remains a challenge, as clinical practitioners need to understand and trust the predictive results of these models. Additionally, issues such as privacy and security need to be fully considered to ensure the protection and compliance of patient data. Looking ahead, it is hoped that with the continuous advancement of medical technology, the application of AI in urologic oncology, including diagnosis, treatment planning, and rehabilitation monitoring, will be further expanded to provide more comprehensive medical services for patients. In conclusion, despite facing challenges, the future prospects of AI in urologic oncology remain promising. Through ongoing research and innovation, AI is expected to provide more personalized and accurate medical services for urologic cancer patients, making a positive contribution to improving patient survival rates and quality of life.
